# Renal Injury in DENV-4 Fatal Cases: Viremia, Immune Response and Cytokine Profile

**DOI:** 10.3390/pathogens8040223

**Published:** 2019-11-07

**Authors:** Priscila Conrado Guerra Nunes, Lilimar da Silveira Rioja, Janice Mery Chicarino de Oliveira Coelho, Natália Gedeão Salomão, Kíssila Rabelo, Carollina Ceia José, Francisco das Chagas de Carvalho Rodrigues, Elzinandes Leal de Azeredo, Carlos Alberto Basílio-de-Oliveira, Rodrigo Basílio-de-Oliveira, Rita Maria Ribeiro Nogueira, Juan Camilo Sánchez-Arcila, Flávia Barreto dos Santos, Marciano Viana Paes

**Affiliations:** 1 Laboratório de Imunologia Viral, Instituto Oswaldo Cruz, Fundação Oswaldo Cruz, Rio de Janeiro-RJ 21040900, Brazil; elzinandes@ioc.fiocruz.br (E.L.d.A.); flaviab@ioc.fiocruz.br (F.B.d.S.); 2 Departamento de Patologia e Laboratórios, Universidade do Estado do Rio de Janeiro (UERJ), Rio de Janeiro-RJ 20550170, Brazil; lilimar.rioja@gmail.com; 3 Laboratório de Anatomia Patológica, Instituto Nacional de Infectologia, Fundação Oswaldo Cruz /FIOCRUZ, Rio de Janeiro-RJ 21040900, Brazil; janice.coelho@ini.fiocruz.br (J.M.C.d.O.C.); francisco.rodrigues@ini.fiocruz.br (F.d.C.d.C.R.); 4 Laboratório Interdisciplinar de Pesquisas Médicas, Instituto Oswaldo Cruz, Fundação Oswaldo Cruz /FIOCRUZ, Rio de Janeiro-RJ 21040900, Brazil; natgsalomao@gmail.com (N.G.S.); carollina.ceia@gmail.com (C.C.J.); 5 Laboratório de Ultraestrutura e Biologia Tecidual, Universidade do Estado do Rio de Janeiro/UERJ, Rio de Janeiro- RJ 20550170, Brazil; kissilarabelo91@gmail.com; 6 Anatomia Patológica, Universidade Federal do Estado do Rio de Janeiro, Rio de Janeiro-RJ 20270004, Brazil; basiliopatologia@br.inter.net (C.A.B.-d.-O.); rodrigopboliveira@gmail.com (R.B.-d.-O.); 7 Laboratório de Flavivirus, Instituto Oswaldo Cruz, Fundação Oswaldo Cruz /FIOCRUZ Rio de Janeiro-RJ 21040900, Brazil; ritanog72@gmail.com; 8 School of Natural Sciences, University of California, Merced-CA 95343, USA; juancamilos@gmail.com

**Keywords:** dengue 4, fatal case, viremia, histopathology, cytokines, inflammatory mediators

## Abstract

Dengue virus (DENV) infections may result in asymptomatic cases or evolve into a severe disease, which involves multiple organ failure. Renal involvement in dengue can be potentially related to an increased mortality. Aiming to better understand the role of DENV in renal injury observed in human fatal cases, post-mortem investigations were performed in four DENV-4 renal autopsies during dengue epidemics in Brazil. Tissues were submitted to histopathology, immunohistochemistry, viral quantification, and characterization of cytokines and inflammatory mediators. Probably due the high viral load, several lesions were observed in the renal tissue, such as diffuse mononuclear infiltration around the glomerulus in the cortical region and in the medullary vessels, hyalinosis arteriolar, lymphocytic infiltrate, increased capsular fibrosis, proximal convoluted tubule (PCT) damage, edema, PCT debris formation, and thickening of the basal vessel membrane. These changes were associated with DENV-4 infection, as confirmed by the presence of DENV-specific NS3 protein, indicative of viral replication. The exacerbated presence of mononuclear cells at several renal tissue sites culminated in the secretion of proinflammatory cytokines and chemokines. Moreover, it can be suggested that the renal tissue injury observed here may have been due to the combination of both high viral load and exacerbated host immune response.

## 1. Introduction

Dengue virus (DENV) has four related but antigenically distinct serotypes (DENV-1 to 4). The viral genome consists of a single RNA strand of positive polarity [1,2] that encodes three structural proteins (C, prM, and E), seven non-structural proteins (NS1, NS2A, NS2B, NS3, NS4A, NS4B, and NS5), and the presence of the non-structural proteins is indicative of viral replication, except for NS1 [3,4,5].

In Brazil, the first DENV-4 cases leading to this serotype spread nationwide, occurring in Roraima and Amazonas in 2010, about 30 years after its first detection in the country [6]. In 2012, DENV-4 was prevalent and detected in 63% of the cases reported in Brazil [7]. Despite the highest notification in 2013 (1,452,289 cases), DENV-4 circulation was associated with mild cases [8,9], but severe and fatal cases due to DENV-4 were also reported [10]. Although there has been no definitive association of the different DENV serotypes within the clinical course of the disease, it has been suggested that DENV-2 and DENV-3 are more frequently associated with severe disease [11]. 

Most patients experience asymptomatic and mild disease; however, a small proportion may evolve to a severe disease, mostly characterized by plasma leakage and hemorrhagic manifestations, which includes multiple organ failure [12].

Several reports suggest an increase in the incidence of dengue-complicated infections affecting different organ systems, such as gastrointestinal, hepatic, respiratory, cardiac, neurological, and renal [13,14,15,16,17,18]. However, studies evaluating renal tissues from dengue fatal cases are scarce. Renal involvement in dengue can potentially be related to an increased mortality, possibly due to indirect effects of the host immunity [19]. Therefore, post-mortem studies on the kidney of dengue-infected individuals may provide important information on the disease immunopathology. In the present study, post-mortem investigations were performed in kidney tissues from DENV-4 fatal cases that occurred during dengue epidemics in Brazil, as the pathogenic mechanisms involved in the severe forms of the disease are still a challenge.

## 2. Results

DENV-4 was the infecting serotype identified and quantified in all kidney tissues by molecular techniques, and the mean viral quantification was 3.34 × 10^9^ copies of RNA/mL (cases 1 to 4 with 5.85 × 10^9^, 4.56 × 10^9^, 1.89 × 10^9^, and 1.06 × 10^9^ copies of RNA/mL, respectively). 

**Figure 1:**
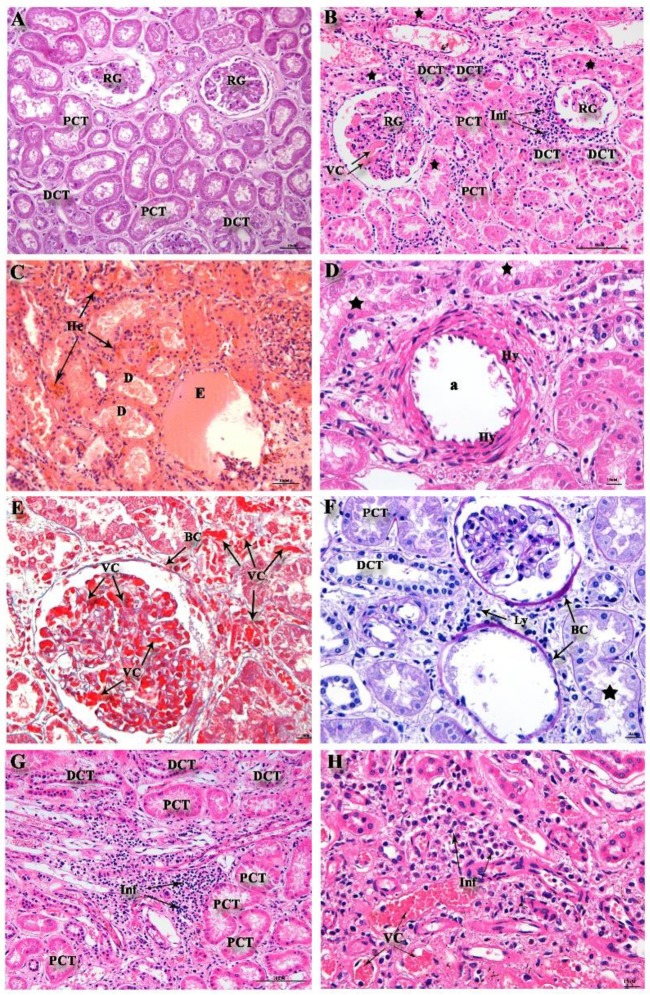
Renal histological analysis. (**A**) Non-dengue control evidencing cortical region with the presence of renal glomerulus (GR) and proximal contiguous tubules (PCT) and distal tubules (DCT). (**B**) Dengue case showing the presence of inflammatory infiltrate (Ly), hyalinoses (Hy) arteriole (a), vascular congestion (VC) in the renal glomerulus (RG), thickened Bowman’s capsule (BC) and damage (star) PCT. (**C**) Dengue case showing tubular debris (D), edema (E), and pockets of hemorrhage (He). (**D**) Presence of hyalinoses (Hy) arteriole (a) and damage (star) PCT in the dengue case. (**E**) Glomerular and peri-glomerular vascular (VC) congestion, presence of Bowman’s capsule (BC), and damage (star) PCT in the case of dengue stained with Masson. (**F**) Peritubular lymphocyte infiltrate (Ly), Bowman’s capsule (BC), damage (star) PCT, and preserved DCT in dengue case stained with Periodic acid-Schiff reaction (PAS). (**G**) Lymphocyte infiltrates (Inf) inside the capillary loops around the proximal convoluted tubules in the cortical region of dengue fatal cases. (**H**) Mononuclear infiltrates (Inf) and vascular congestion (VC) around collecting tubes in the medullar region of dengue fatal cases.

The histopathological analysis showed vascular congestion of glomerular capillary and medullar region in all kidney tissues (Figure 1B,E,H). As expected, in the kidney of non-dengue patients, a regular structure of the glomerulus and normal structure with preserved distal and proximal convoluted tubules were observed (Figure 1A). Isolated glomerular obsolescence (Figure 1B,F) and focal inflammatory infiltrate in the cortical (Figure 1B,F) and medullary region (Figure 1G,H) were present in case 1. In the proximal convoluted tubules (PCT), loss of the apical cell portion and cytoplasmic remains (star) in light were observed in cases 2 and 4 (Figure 1D,F), and congestion of peritubular capillaries was found in cases 1, 2, and 4 (Figure 1B,E,H). Discrete focus of isolated hyalinosis was observed in case 1 and case 3, showing a prominent arteriolar hyalinosis (Figure 1D). The glomeruli presented visceral epithelial cells and tubular cells at confluence (Figure 1B), as well as hyperplastic epithelial cells in the cortical region (Figure 1F) in case 1. In addition to the changes described in the renal glomerulus, a focus of interstitial fibroedema and tubular atrophy with discrete mononuclear inflammatory infiltrate, focal cell necrosis, granular material in the fibrous endarteritis lumen into the intertubular arteries, and focal hemorrhage were observed only in case 3 (Figure 1C). 

**Figure 2:**
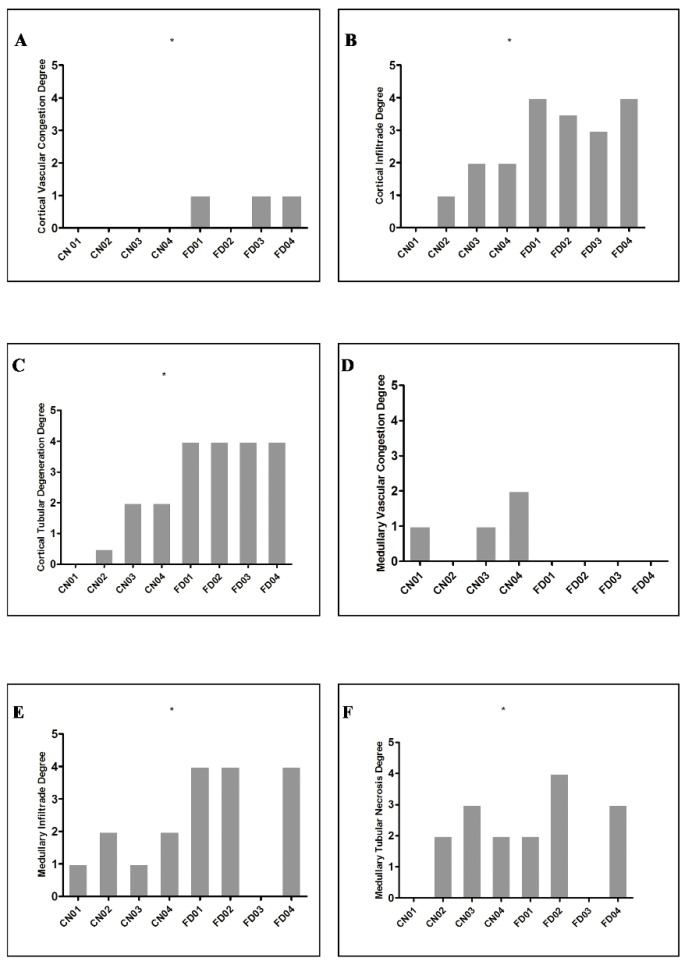
Median quantification of renal damage in four dengue fatal cases (*n* = 4) compared to control cases (*n* = 4). (**A**) Vascular congestion in renal cortical regions (*p = 0.0040*). (**B**) Tubular degeneration (*p* ≤ *0.0001*). (**C**) Renal cortical infiltrates (*p* ≤ *0.0001*). (**D**) Vascular congestion in renal medullary regions (*p = 0.1280*). (**E**) Tubular necrosis (*p = 0.0008*). (**F**) Infiltrates in renal medullary regions (*p* ≤ *0.0001*).

The analysis on the damage between fatal and non-fatal cases showed a significantly higher damage in the cortical and medullary region in the fatal cases as compared to the control cases (see Table 1 and Figure 2). 

**Figure 3:**
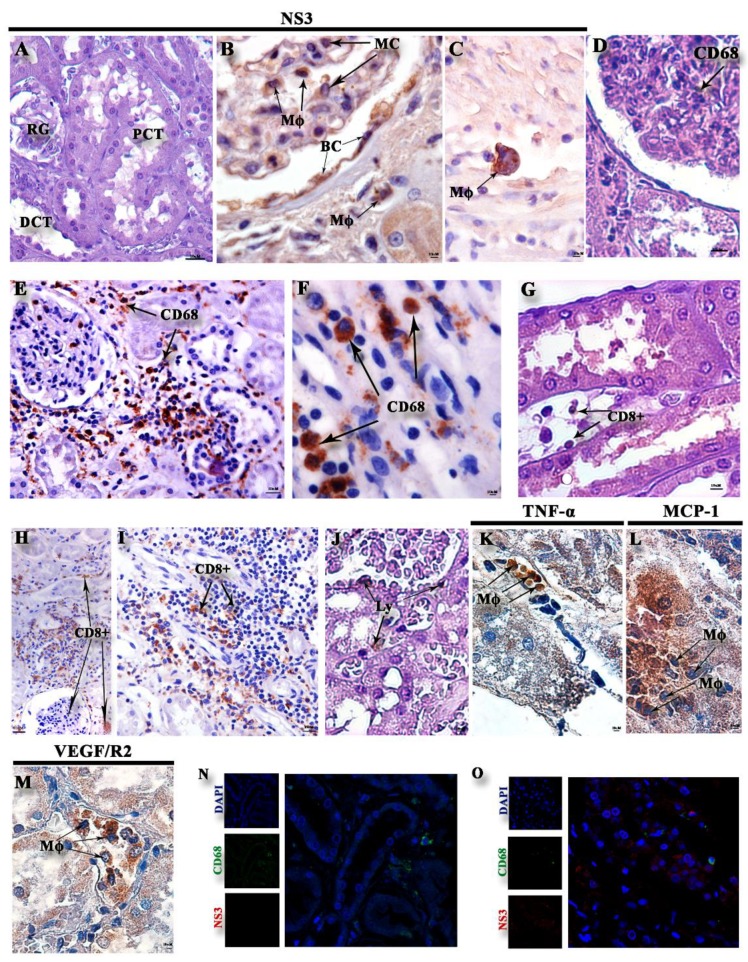
Immunohistochemistry of viral staining, subpopulations, and cytokines/chemokines: (**A**) Non-dengue case control without dengue virus (DENV) NS3 staining. (**B**,**C**) Detection of DENV NS3 in the dengue case within macrophages (Mø), Mesenchymal cells (MC), endothelial cells (En) in the cortical region, and macrophages (Mø) in the medullar region. (**D**) Control with CD68 staining inside the glomerulus. (**E**,**F**) Expression of CD68-labeled cells in the cortical and medullary regions of the dengue case. (**G**) Control with CD8^+^ peritubular staining. (**H**,**I**) Expression of CD8^+^ T cells in the medullary and cortical region of the DENV-4 case. (**J**) Representative negative control of cytokine and inflammatory mediators. (**K**) DENV-4 case with mononuclear infiltrate (Inf) expressing TNF-α, macrophages (Mø) expressing MCP-1 (**L**), and macrophages (Mø) expressing VEGF/R2 (**M**). Cells showing double staining (green and red) were observed in control (**N**) and fatal dengue cases (**O**).

Immunohistochemistry assay demonstrated the presence of DENV NS3 protein in macrophages and mesangial cells in the renal glomerulus (Figure 3B), parietal leaflet of the Bowman’s capsule (Figure 3B), and in isolated macrophages of the cortical region (Figure 3B). Compared to the case control, there was an increase in the CD68 cellular infiltration (Figure 3E,F) and CD8+ T cells (Figure 3H,I) in the cortical and medullary regions. There was also an increase of TNF-α in the peritubular mononuclear cells infiltrate (Figure 3K), an increase of macrophages expressing Monocyte chemoattractant protein-1 (MCP-1) in the capillary peritubular region (Figure 3L), and an increase in Vascular endothelial growth factor receptor 2 (VEGF/R2) expression in peritubular macrophages in the medullary region (Figure 3M). DENV NS3 and CD68+ double-stained cells were also observed (Figure 3O).

## 3. Discussion

DENV can infect several cell types in different tissues and organs. Studies in autopsies and biopsies of patients have demonstrated viral presence in monocytes and macrophages in the liver, lung, spleen, brain, kidney, bone marrow, and heart [20,21,22,23,24].

Dengue renal involvement varies from elevated serum creatinine, acute tubular necrosis, hemolytic uremic syndrome, proteinuria, glomerulopathy, nephrotic syndrome, and acute renal injury [25]. Despite the remarkable evidence that the transient increase in serum creatinine is linked to increased mortality [26], acute renal injury is a poorly studied complication in dengue. The available data indicate the presence of acute renal injury occurs in 0.83% to 14.2% of dengue patients, depending on the methodology and the population evaluated [18,27,28,29,30,31,32].

Kidney damage can be induced by viral infection, in which a direct viral cytopathic effect may occur on glomerular and tubular cells. An immune-mediated in situ mechanism triggered by viral antigens in the glomeruli can cause tissue damage and deposition of immunocomplexes and antiviral antibodies, and the expression of inflammatory mediators are released in response to exacerbated inflammation in the intertubular vessels [33]. Analysis of autopsies or biopsies of human cells infected by DENV using immunohistochemistry and in situ hybridization techniques detected viral antigens on tubular epithelial cells [21,23,34]. 

Jessie et al. [21] analyzed human tissues from DENV-1 infected patients and found viral antigens as discrete granular deposits within the tubule-lining cells, but found no viral RNA in the samples. Here, on the other hand, high viral titers in all DENV-4 cases were observed. Basilio-de-Oliveira et al. [23] demonstrated in a fatal elderly DENV-3 case, hemorrhage in the glomerular capillaries, and proximal convoluted tubules. Renal medullary tissue also presented a mononuclear infiltrate around the collection ducts, with pockets of hemorrhage, interstitial edema, and vascular congestion. Similarly, our cases presented more diffuse and focal infiltrates. Moreover, congestion, peritubular hemorrhage in case 3, and congestion in all cases, both in the medullary and in the cortical area, were identified.

Histological analysis revealed circulatory and parenchymal damage, presenting acute tubular necrosis, characterized by desquamation of necrotic cells and loss of the basal membrane mainly in contorted proximal tubules, thrombotic microangiopathy, and glomerulopathy [20,35,36,37,38]. Studies have reported immune complex-mediated lesion in the glomerulus in patients with DENV infections, and have suggested this as a possible mechanism for urinary abnormalities [39,40]. The histopathological changes observed here exhibited a characteristic lesion of tubular necrosis, seen in cases with co morbidities, such as in diabetes [41].

In three DENV-3 fatal cases, Póvoa et al. [20] described the presence of acute tubular necrosis, characterized by desquamation of necrotic cells and loss of the basal membrane, mainly in contorted proximal tubules but also, to a lesser extent, in the distal tubules, with mold formation of cellular debris, similarly to what has been observed in the DENV-4 cases analyzed here, suggesting that renal damage caused by DENV is not serotype-specific.

In this study, we observed that the presence of vascular congestion around and within the glomerulus in the cortical and medullary region, arteriolar hyalinosis, damaged mesangial cells, lymphocytic infiltrate in the cortical and medullary region, fibrous increase in the Bowman’s capsule, and thickened basement membrane in the cortical region are all associated with DENV-4 infection and replication, demonstrated by the presence of the DENV NS3 protein [20,42]. 

Arteriolar hyalinosis occurs by glomerular hyperfiltration [43], and this lesion is not exclusive to a specific disease, being observed in arterioles of normal individuals with advanced age [44]; however, it may occur earlier and more intensely in cases of hypertension and diabetes mellitus. However, this is the first study to describe this renal alteration in young adult fatal cases being caused by serotype 4.

An inflammatory environment with mononuclear cells in several sites in the renal tissue was observed in this study. The evaluation of the immune response in the renal and medullary cortex showed increased detection of macrophages (CD68+) and CD8+ T cells in the peritubular space and around vessels in the medullary. CD68+ cells showed their co-expression with the DENV NS3 protein, confirming the participation of those cells during DENV-4 infection in renal tissue. In addition, the migration of CD68+ and CD8+ T cells could indicate those cells playing a role in the cytotoxic response in such lesions as a way to contain viral infection, leading to secretion of proinflammatory cytokines and chemokines [45] in the cortical and medullary regions of the kidney. Furthermore, it may be suggested that the renal tissue injuries observed here may have been due to the combination of both high viral load identified in those tissues and the exacerbated host immune response.

Dengue may cause vascular overload and reduction of intravascular volume. This may result in the reduction of renal perfusion leading to acute tubular necrosis, and involvement of multiple organ failure in severe dengue [34,46,47]. Moreover, intense capillary leakage may lead to multiple organ dysfunction [48] and those observations corroborate the changes observed in other organs of those cases, as systemic lesions in several tissues were observed (data not shown).

Retrospective case series studies have demonstrated that acute renal injury induced by dengue is a highly morbid and fatal complication and is associated with prolonged hospitalization [30,32]. However, it is known that the present changes observed here cannot be analyzed isolated, as the changes in dengue are dynamic and the virus can be disseminated to all vital organs of the host. However, studies such as the one presented here may be useful as an alert so that the identification of renal injury parameters can be observed in dengue cases and so that proper management is performed.

This study has some limitations, including the quality of the record resulting in the lack of some clinical information.

## 4. Materials and Methods 

### 4.1. Ethical Considerations

The samples used in this study were received as convenience samples at the Pathological Anatomy Laboratory, National Infectology Institute (Instituto Nacional de Infectologia Evandro Chagas/INI), FIOCRUZ and were investigated in a collaboration with the Flavivirus Laboratory, Oswaldo Cruz Institute, IOC, FIOCRUZ, Regional Reference Laboratory for the Brazilian Ministry of Health, in a study approved by the research ethics committee (CEP 274/05 and CAAE: 57221416.0.1001.5248) of the Oswaldo Cruz Foundation, Ministry of Health, Brazil.

### 4.2. Dengue Fatal Cases

Young adults (20–33 years old) fatal cases (*n* = 4), were investigated as dengue-suspected cases during epidemics occurring in 2012 and 2013 in Brazil. All cases died within 3 to 4 days after the fever onset and no comorbidity was reported. All cases were confirmed as dengue by a positive result on the tissue by immunohistochemistry and RT-PCR that confirmed DENV-4 as the infecting serotype.

Case 1: A 27 years old female patient, living in Manaus, in the north region of Brazil, presenting fever, myalgia, bleeding, and headache. Leptospirosis was investigated and presented a negative result. Death occurred in January of 2012. 

Case 2: A 22 years old male patient, a resident of Pernambuco, Northeast Brazil, presenting fever with back and lower limb pain, vomiting with blood, respiratory changes, and was quite agitated. This evolved into cardiorespiratory arrest, and doctors initiated a resuscitation maneuver and performed ortho-tracheal intubation. Intubation showed large amounts of blood from the lower airways. According to family members, the patient had a history of fever and lower back pain for 3 days. Leptospirosis was investigated and presented a negative result. Death occurred in 2013.

Case 3: A 33 years old female resident of Mato Grosso do Sul, Midwest region of the country, presenting petechia, metrorrhagia, signs of hemorrhagic shock, lowering of consciousness level, and hemorrhagic shock. She died 4 days after the onset of the symptoms in 2012. Leptospirosis was investigated and presented a negative result.

Case 4: A 20 years old male patient resident of Pernambuco, presenting a persistent cough with hemoptysis for 4 days, which evolved into massive hemoptysis. There was diffuse pulmonary hemorrhage and unspecified hemorrhagic syndrome, dyspneic wheezing, and snoring. Thorax X-ray with eradication of two-thirds of the lung and acute exacerbation (AE) of one-third. The patient died 4 days after the onset of the symptoms, in 2013. Leptospirosis was investigated and presented a negative result. 

### 4.3. Dengue Molecular Diagnosis, Histopathological Analysis, and Immunohistochemistry

Kidney tissues samples from necropsy were paraffin-embedded, fixed in 10% formalin, cut (4 µm), deparaffinized in xylene, and rehydrated with alcohol, as described elsewhere [20]. For the paraffin-embedded viral RNA extraction, three 5 μm slices of each fragment were used and submitted separately to the PureLink FFPE RNA Isolation Kit (Invitrogen, CA, USA). The conventional reverse transcriptase polymerase chain reaction (RT-PCR) for DENV identification and serotyping was performed as described by Lanciotti et al. [49]. DENV-4 quantification was performed by real-time RT-PCR technique described by Johnson et al. [50] using a Taqman quantitative Real Time RT-PCR system. Kidney tissues sections (5 mm thick) were treated with different stains (hematoxylin–eosin, Masson’s trichrome, or PAS). After staining, sections were examined and visualized by light microscopy (Olympus, Tokyo, Japan) and digital images were obtained using Image Pro Plus software version 4.5. Four types of damage were considered for quantification: tubular degeneration in the cortical region, medullary tubular necrosis, infiltrates, and vascular congestion in the medullary region. Tissue damages were quantified and qualified in each of the 15 images as 0—absent, 1—light and focal, 2—light, 3—moderate, and 4—diffuse. To analyze differences in the number of damage counts between fatal and non-fatal cases, we used generalized linear models (GLM) and we built a different model for each tissue region/type of alteration. Differences between fatal and non-fatal cases were reported as *p*-values. This analysis was performed using the *R* statistical environment [51]. For immunoperoxidase assay, antigen retrieval was performed by heating the tissue in the presence of EnVision Flex target retrieval solution high pH (Dako, CA, USA) or citrate buffer. Tissues were blocked for endogenous peroxidase with 3% hydrogen peroxidase in methanol and rinsed in Tris-HCl (pH 7.4). To reduce non-specific binding, sections were incubated for 30 min at room temperature. Samples were then incubated overnight at 4 °C with anti-DENV NS3 recombinant antibody [20], mouse anti-human CD8 Clone C8/144B (Dako, CA, USA), macrophage antibody CD68 clone EBM11 (Dako, CA, USA), anti-MCP 1 monoclonal antibody (Novus Biologicals, CO, USA), anti-TNF alpha antibody, Clone ab6671 (Abcam, MA, USA), Rb anti-FLK-1, and VEGF/R2 (Spring Bioscience, CA, USA). On the next day, the sections were incubated with REVEAL COMPLEMENT secondary antibody (Spring Bioscience, CA, USA) for 10 min, and a REVEAL-HRP secondary antibody conjugate (Spring Bioscience, CA, USA) for 15 min at room temperature. Reaction was revealed with diaminobenzidine (Spring Bioscience, CA, USA) as chromogen and sections were counterstained on Harris hematoxylin (Dako, CA, USA). For immunofluorescence and CD68/NS3 co-staining, the paraffin-embedded tissues were cut at 4 μm and the slides were stained overnight at 4 °C with anti-DENV NS3 recombinant antibody [20] and anti-human monoclonal CD68 (Dako, CA, USA). Sections were incubated with Alexa 488 conjugated rabbit anti-mouse IgG, goat anti-rabbit IgG conjugated to Alexa 555, or goat anti-mouse IgG conjugated to Alexa 555 (ThermoFisher, USA) and analyzed using a Zeiss LSM 510 Meta confocal microscope (Carl Zeiss, Oberkochen, Germany).

## Figures and Tables

**Table 1:** Quantification of damage in kidney regions from four dengue fatal cases.

Region	Injury	Control	Cases	*p*-Value
Cortical	Vascular congestion	0(0–0)	1(0–1)	*0.0040*
	Degeneration	1(0–2)	4(4–4)	*<0.0001*
	Infiltrates	2(0–2)	3(2.75–4)	*<0.0001*
Medullary	Vascular congestion	1(0–2)	0(0–1)	*0.1280*
	Infiltrates	2(1–2)	4(3–4)	*<0.0001*
	Necrosis	2(0–3)	4(2–4)	*0.0008*
